# Hemorrhagic Shock from a Duodenal Ulcer Eroding into an Ectopic Varix

**DOI:** 10.1155/2023/7094924

**Published:** 2023-12-31

**Authors:** Harjit Singh, Alexandra Kimchy, Camille Boustani, Mfoniso Umoren, Amol Rangnekar, Coleman Smith

**Affiliations:** ^1^Department of Internal Medicine, MedStar Georgetown University Hospital, Washington, DC, USA; ^2^Department of Gastroenterology, MedStar Georgetown University Hospital, Washington, DC, USA; ^3^Department of Transplant Hepatology, MedStar Georgetown University Hospital, Washington, DC, USA

## Abstract

Ectopic varices are an infrequent yet fatal complication resulting from the progression of liver cirrhosis. Duodenal varices pose a significant challenge to clinicians as they are not easily visualized on endoscopy due to their submucosal location and lack of red color signs. Identification of duodenal varices is important given the risk of massive and life-threatening bleeding that is difficult to control. Patients may present in hemorrhagic shock requiring immediate resuscitation; however, confirmation of the bleeding source as variceal or non-variceal is critical in determining the optimal therapeutic intervention. Here, we report an unusual case of a duodenal ulcer that eroded into an ectopic varix resulting in hemorrhagic shock.

## 1. Introduction

Ectopic varices are a rare complication of cirrhosis with an estimated prevalence of <3% [1].

Duodenal varices (DV) in particular have a high mortality rate from bleeding at approximately 40%, and thus establishing an early diagnosis is essential to improve patient outcomes [1]. Due to their small diameter and deep location, DV pose a diagnostic challenge endoscopically. They may initially present as thickened duodenal folds with increasing prominence closer to bleeding [2]. In patients with cirrhosis, classification of the bleeding source is further complicated by the additional risk of non-variceal bleeding most commonly due to peptic ulcer disease [2]. Given its anatomic location, the gastroduodenal artery is the vessel typically associated with bleeding from posterior wall duodenal bulb ulcers [3]. Effective management of upper gastrointestinal bleeding in cirrhotic patients is dependent upon accurately identifying of the origin of bleeding prior to intervention [4, 5]. Here, we report a rare case of a duodenal ulcer that eroded into an ectopic varix resulting in hemorrhagic shock.

## 2. Case Report

**Figure 1:**
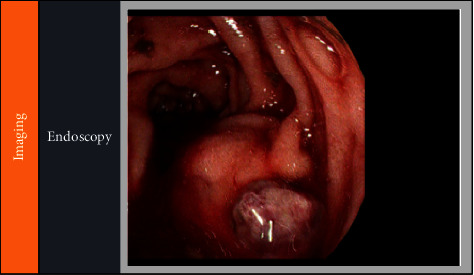
Endoscopy demonstrating a single ulcer with an adherent clot and active bleeding.

**Figure 2:**
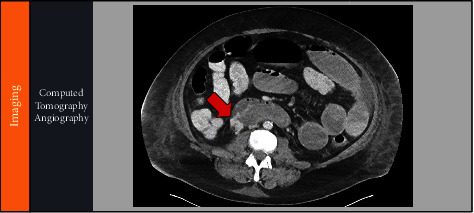
Computed tomography angiography showing a large duodenal varix with active hemorrhage into the second portion of the duodenum (red arrow).

A 47-year-old male with a history of decompensated alcohol-related cirrhosis with ascites (MELD-Na score of 40) and seizure disorder was transferred to our liver transplant center's medical intensive care unit for management of hemorrhagic shock secondary to an upper GI bleed. On arrival to our center, the patient was hypotensive requiring continuation of vasopressors. He had large volume hematochezia with an associated drop in Hgb to 4.3 g/dL requiring initiation of the massive transfusion protocol, ultimately receiving 9 units of packed red blood cells, 6 units of fresh frozen plasma, 4 units of cryoprecipitate, and 6 units of platelets. After initial resuscitation, gastroenterology was consulted for an emergent esophagogastroduodenoscopy given the concern for a brisk upper gastrointestinal bleed. The endoscopy showed no obvious esophagogastric or duodenal varices, but mild portal hypertensive gastropathy, 3 non-bleeding, clean-based ulcers in the duodenal bulb, and 1 ulcer with an adherent clot that was actively bleeding into the 2^nd^ portion of the duodenum (Figure 1). A submucosal 10 mL epinephrine injection was performed around the bleeding site to achieve hemostasis. Given the high-risk for rebleeding, the patient underwent celiac and superior mesenteric angiography, which showed no active extravasation. Empiric coil embolization of the gastroduodenal artery was performed by interventional radiology (IR). Two days following the procedure, the patient developed worsening hypotension with new hematemesis requiring additional vasopressors and blood products. He underwent a computed tomography (CT) angiography of the abdomen and pelvis, which showed active hemorrhage into the 2^nd^ and 3^rd^ portion of the duodenum from a large duodenal varix extending into the eroded duodenal wall (Figure 2). No portal vein thrombosis was noted on CTA. In discussion with IR, there was no clear route to access the duodenal varix endovascularly for embolization. Furthermore, due to his worsening coagulopathy and hemodynamic instability, the patient was not a candidate for an emergent transjugular intrahepatic portosystemic shunting (TIPS) procedure. Given his poor prognosis, the patient's family decided to pursue comfort care measures, and he shortly succumbed to his illness.

## 3. Discussion

In patients with cirrhosis, it can be difficult to determine the etiology of an upper GI bleed due to the presence of large portosystemic venous collaterals in the setting of portal hypertension [1]. Ectopic varices located in the duodenum are rare but when bleeding occurs, it is often life-threatening [1]. Identification of duodenal varices (DV) as the source of bleeding requires thorough examination of the duodenal bulb and 2^nd^ through 4^th^ parts of the duodenum with serial endoscopies [2]. Endoscopy has been shown to be effective in identifying DV in only 44% of cases, and thus there is need for additional diagnostic modalities [2]. CT imaging has demonstrated some utility in establishing pathways of portal collateral circulation; however, endoscopic ultrasound (EUS) has been shown to be more sensitive for detection of DV. A case series by Sharma et al. found that EUS was able to detect the presence of DV with superior accuracy compared to conventional endoscopy alone [2]. Furthermore, EUS was better able to delineate duodenal perforators than traditional angiography [2]. Identification of duodenal perforators is critical, as ligation is associated with decreased rates of hemorrhage [2]. However, further studies are needed to assess the effectiveness of non-endoscopic diagnostic modalities in detecting DV.

Treatment of DV is aimed at occlusion of the portal venous perforators as opposed to arterial embolization used in non-variceal, peptic ulcer bleeding. The current therapeutic options include endoscopic varix band ligation (EVL), TIPS, percutaneous transhepatic obliteration, balloon-occluded retrograde transvenous obliteration, and surgery [5, 6]. However, given the scarcity of literature, there is a lack of consensus regarding a standard treatment approach for DV. The most common therapeutic intervention is EVL, although the rebleeding risk remains high due to the minimal thickness of the duodenal wall [6]. One study evaluating the effectiveness of EVL for the treatment of DV found the overall success rate to be low at approximately 16% [6]. Results from recent studies investigating the efficacy of sclerotherapy with injection of polidocanol for management of DV have been promising; however, additional large-scale studies are needed to further assess the long-term viability as treatment [6].

Despite their rarity, the mortality rate of bleeding DV is high. Success of treatment is multifactorial and depends on patient characteristics as well as operator and center expertise [6]. Therefore, it is imperative that patients with cirrhosis and upper gastrointestinal bleeding undergo a comprehensive diagnostic evaluation. Our case highlights the importance of differentiating between variceal and non-variceal bleeding in determining the optimal therapeutic approach to achieve hemostasis. Physicians should remain vigilant for ectopic varices in patients with cirrhosis and peptic ulcer disease due to the risk of variceal bleeding [7].

## Data Availability

No data were used to support this study.
